# Effects of hyperbaric oxygen therapy on recovery after a football match in young players: a double-blind randomized controlled trial

**DOI:** 10.3389/fphys.2024.1483142

**Published:** 2024-10-22

**Authors:** Marko Gušić, Tomislav Stantić, Anja Lazić, Slobodan Andrašić, Bart Roelands, Špela Bogataj

**Affiliations:** ^1^ Faculty of Sport and Physical Education, University of Novi Sad, Novi Sad, Serbia; ^2^ Faculty of Sports and Tourism, Educons University, Novi Sad, Serbia; ^3^ Faculty of Sport and Physical Education, University of Niš, Nis, Serbia; ^4^ Faculty of Economics, University of Novi Sad, Novi Sad, Serbia; ^5^ Human Physiology and Sports Physiotherapy Research Group, Vrije Universiteit Brussel, Brussels, Belgium; ^6^ Brussels Human Robotics Research Center (BruBotics), Vrije Universiteit Brussel, Brussels, Belgium; ^7^ Department of Nephrology, University Medical Centre Ljubljana, Ljubljana, Slovenia; ^8^ Faculty of Sport, University of Ljubljana, Ljubljana, Slovenia

**Keywords:** hyperbaric oxygen therapy, football, performance, recovery, young athletes, blood analysis

## Abstract

**Introduction:**

Football is a physically demanding sport that requires effective recovery strategies to maintain performance level and prevent injuries. This study investigated if a single 1-h hyperbaric oxygen therapy (HBOT) session affects recovery and performance after a football match in elite youth players.

**Methods:**

Twenty elite youth football players (age 17.3 ± 0.5 years) were randomly assigned to a HBOT group or a control group (CON). They played a 90-min football game and underwent either a 60-min HBOT or placebo intervention. Before (T1), at the end of the match (T2), 1 h after HBOT or CON session (T3), and 12 h after HBOT session (T4), subjects underwent biochemical (serum samples (myoglobin (MB), creatine kinase (CK), lactate dehydrogenase (LDH), alanine aminotransferase (ALT), and aspartate aminotransferase (AST)) and performance measurements (linear speed at 5 m, 10 m and 20 m, squat jump (SJ), countermovement jump (CMJ) and countermovement jump with arm swing (CMJa)). The Hooper Index (HI) was collected and heart rate was measured during the game.

**Results:**

The football match induced significant increases in all biochemical markers, but no significant differences were found between the HBOT and control group in biochemical or performance parameters at any time point. However, there was a significant interaction effect between time and group for HI (*p* = 0.012, η2 = 0.124), with the HBOT group showing significantly lower HI values (8.6 ± 2.41) than the control group (11.0 ± 3.23) at 1 h post-HBOT.

**Discussion:**

A single 1-h session of HBOT did not significantly affect recovery or performance parameters in elite youth football players, though it did show a moderate positive affect on the HI at 1 h post-HBOT. Further studies should explore the impact of either longer or sequential HBOT sessions on recovery.

## 1 Introduction

Football is a physically demanding, competitive sport that causes stress on various physiological systems (Reilly and Ekblom, 2005). Previous studies (Silva et al., 2013; Viana-Gomes et al., 2018) showed that a single match causes significant increases in oxidative stress and cellular damage indicators in the plasma of professional football players. This can lead to acute, albeit usually small, decrements in sprint and jump performance (Abaïdia and Dupont, 2018). The tight schedule, the intermittent nature of the sport, performing muscle-damaging actions, and limited time for recovery can lead to an increased risk of fatigue, delayed-onset muscle soreness (DOMS) and injuries (Barnett, 2006; Cheung et al., 2003). In addition, previous studies (Abaïdia and Dupont, 2018; Magalhaes et al., 2010) reported that complete recovery after one match requires up to 72 h. Therefore, effective and fast recovery is the key to uphold performance levels.

Considerable research attention has been given to strategies to accelerate acute recovery in football players. Nutrition and antioxidant supplements, sleep, cold water immersion, active recovery, and compression garments have been suggested (Dupuy et al., 2018; Nédélec et al., 2013; Santos et al., 2016) to reduce anti-inflammatory responses and fatigue immediately after the match, as well as to prevent further functional impairment. However, the area of effective recovery strategies remains unclear due to the limited number of studies that investigated elite players (Barnett, 2006) and the variety of modalities without empirical evidence of which one should be followed. Since the literature indicates the essential role of fast recovery, but lacks clear findings (Kellmann, 2010), an increased interest in hyperbaric oxygen therapy (HBOT) has emerged in recent years (Mihailovic et al., 2023). HBOT has been reported to accelerate cell regeneration and tissue repair, which should help eliminate fatigue and restore endurance capacity. More precisely, HBOT is a treatment in which 100% oxygen is supplied under elevated pressure. Such treatment increases dissolved oxygen levels in the blood and results in a high partial pressure of oxygen in peripheral tissues, which is beneficial for conditions associated with low oxygen environments, potentially stimulating the recovery process (Hodges et al., 2003). HBOT can improve the oxygenation of skeletal muscles, which accelerates the production of adenosine triphosphate (ATP) in addition to the metabolic purification of metabolites that cause fatigue (Sperlich et al., 2017). Moreover, HBOT is proven to be safe and effective in the non-athletic population as a treatment for accelerating the healing process and reducing local hypoxia and inflammation (Moghadam et al., 2020). Until now, HBOT has been applied in various ways, ranging from acute (single) sessions, such as 60-min interventions (Park et al., 2018; Branco et al., 2016), to more chronic protocols involving multiple sessions per week over extended periods (Hadanny et al., 2022; Mihailovic et al., 2023). The choice of protocol often depends on the goals, such as acute recovery or long-term adaptation.

HBOT administration has been proposed as an adjuvant treatment for improving muscle repair and recovery from exercise-induced muscle damage (Ishii et al., 2005). Surprisingly, although oxygen has the most important role during recovery, only two studies (Branco et al., 2016; Mihailovic et al., 2023) have investigated the post-exercise impact of HBOT on biochemical recovery parameters or performance in athletes. Branco et al. (2016) investigated the effects of a single session HBOT on hormonal and cell damage markers in professional jiu-jitsu athletes and failed to find a significant influence of HBOT, while Park et al. (2018) found positive effects of a single 60-min HBOT on the removal of peripheral fatigue symptoms induced by maximal exercise in college football players. Similarly, in terms of the acute effects of HBOT on performance, previous studies (Hadanny et al., 2022; Hodges et al., 2003; McGavock et al., 1999; Mihailovic et al., 2023) indicated equivocal findings. While early studies (Hodges et al., 2003; McGavock et al., 1999) suggested no effects of a single HBOT session on performance, later research (Mihailovic et al., 2023; Park et al., 2018) seems to indicate that HBOT was able to improve cardiac parasympathetic reactivation by HBOT applications over several weeks and recovery of peripheral fatigue by a single HBOT session.

The role of HBOT on recovery parameters and performance in elite football players is still poorly understood, and to date, no study has specifically examined the effects of a single session of HBOT after a football match. Since football is an intermittent sport with high physical, physiological, and metabolic demands, HBOT might be considered a promising post-exercise recovery treatment. Therefore, this study aimed to investigate the effects of a single HBOT session on recovery and performance after a football match in elite youth football players. We hypothesized that a single session of HBOT would significantly enhance recovery and improve performance compared to a placebo condition.

## 2 Methods

### 2.1 Participants

Twenty elite youth male football players (age: 17.3 ± 0.5 years; football experience: 10.2 ± 1.7 years) participated in the study, and informed consent was obtained from their parents or legal guardians. Participants were free of injuries and medical conditions contraindicated by HBOT. All participants were informed of the study procedures and provided written parental consent prior to participation. Participants were advised to refrain from severe physical activity for 24 h and to abstain from breakfast, caffeine, and alcohol prior to the first blood collection.

### 2.2 Study design and procedures

**FIGURE 1:**
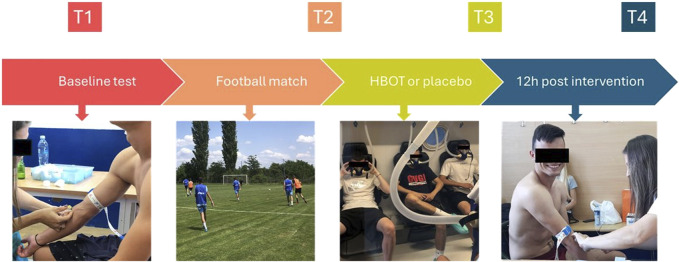
Study design.

The study was conducted using a randomized, double-blind design. The participants were randomly assigned to either the HBOT group or the control (CON) group. All participants were evaluated for biochemical parameters, physical performance tests and Hooper index (HI). The participants were tested four times (Figure 1): at baseline, pre-match (T1), at the end of the match, post-match (T2), 1 hour after the HBOT session (T3), and 12 h after the HBOT session (T4). Fasted blood samples were collected before breakfast. After a standardized light meal and rest period, participants were evaluated for physical performance with the linear speed and vertical jump (VJ) height tests before playing a football match (T1). Participants were randomized in a 1:1 fashion into two groups based on their playing positions to play against each other. In the afternoon the match took place on an outdoor football field and lasted for 90 min at an average ambient temperature of 26°C. It was a simulated game replacing a regular training session, with 22 players. Both teams used a 5-three to two line up. Goalkeepers were excluded from the analysis because of the different physical demands of this position. The same examination was performed after the match (T2), followed by HBOT or CON treatment. Finally, blood samples and physical fitness were evaluated 1 h (T3) and 12 h (T4) after the intervention. In addition, heart rate (HR; Polar Team System H7 (Polar Electro Oy, Kempele, Finland)) was constantly tracked during the match to determine its intensity. Procedures were conducted with the requirements and approval of the Ethical Committee of Faculty of Sport and Physical Education, University of Niš (ref. 04–651/2; approval date 23 May 2022) and registered at ClinicalTrials.gov under NCT06112210.

### 2.3 Biochemical analysis

Blood samples were collected from the antecubital vein with participants in a seated position. A vacutainer tube was used to collect blood samples (Vacusera 5 mL Serum Gel and Clot Activator, Disera A.S. Izmir-Turkey) and centrifuged immediately at 3,000 rpm for 10 min to isolate the serum. Myoglobin (MB) was determined with Siemens IMMULITE 1000 (Siemens Healthcare Diagnostics, UK), while creatine kinase (CK), lactate dehydrogenase (LDH), alanine aminotransferase (ALT), and aspartate aminotransferase (AST) were analyzed using a biochemistry analyzer (A25 Biosystems Chemistry Analyzer).

### 2.4 Vertical jump (VJ) height

Squat jump (SJ), countermovement jump (CMJ) and countermovement jump with arm swing (CMJa) tests were performed to measure the VJ height. The Optojump system (Microgate, Bolzano, Italy) was used to determine vertical VJ height; its validity and reliability had previously been shown (Glatthorn et al., 2011). Previously, the procedure as well as the validity and reliability of the tests in football players were examined (Claudino et al., 2017; Garrett et al., 2020). Briefly, the SJ was executed in a semi squat position with the knees bent at 90° and the arms resting on the hips. Participants held this stance for one to 3 seconds before the maximal VJ. Moreover, the CMJ and the CMJa were evaluated while standing straight up, with equal weight distributed over both feet. The hands were free to move when the CMJa was conducted. Furthermore, the arms were secured to the hips during the CMJ to prevent their impact on jump height. Afterwards, participants squatted down to a 90-degree flexion and executed a maximal VJ without pausing at the moment of direction change. Each participant was provided with instructions to perform all jumps with maximal effort. Furthermore, each jump was attempted three times, with the best result being included in the statistical analysis.

### 2.5 Linear speed

Linear speed was measured from a standing position at 5 m, 10 m, and 20 m, as previously described (Buchheit et al., 2010; Dugdale et al., 2019). The Witty photocell system (Witty, System, Microgate, Bolzano, Italy) was used to assess the performance. In addition, to reduce the influence of the hand swing when passing through the gate, photocells were positioned at 10 m and 20 m from the starting line, 0.4 m above the ground, and with an accuracy of 0.001 m/s (Yeadon et al., 1999). Participants were instructed to exert full effort when passing through all gates.

### 2.6 Hyperbaric oxygen therapy (HBOT) procedure

Both the HBOT and placebo protocols were performed in a Barox HBOT chamber (Yaklasim Makina San. Ve Tic. Ltd. Sti). The participants were taken to the chamber immediately after the football match, where they sat in individual chairs and oxygen was delivered through individual masks. The HBOT group was exposed to 100% oxygen at 2.2 ATA (atmospheres absolute), while the CON group was exposed to normobaric ambient pressure (1 ATA). Both sessions lasted 60 min. This protocol was selected to follow current literature (Babul et al., 2003; Mekjavic et al., 2000; Shimoda et al., 2015; Webster et al., 2002).

### 2.7 Hooper index (HI)

HI was used for the subjective assessment of fatigue and players’ wellbeing. Subjects were unfamiliar with the HI and therefore it was thoroughly explained before the start of the experiment. The HI is a summation of the four subjective ratings: sleep (concerning the night preceding the evaluation), fatigue, stress and DOMS on a scale of 1–7 (Hooper et al., 1995). Specifically, ‘1’ represents “very, very good state”, and ‘7’ represents “very, very bad state”. After the participants completed the questionnaire, the HI was calculated and used for analysis. Moreover, HI has been shown to be a valid and reliable tool for monitoring fatigue in professional football players (Clemente et al., 2017; Thorpe et al., 2015).

### 2.8 Data analysis

Data analysis was performed with the Statistical Package for the Social Sciences (v29.0; SPSS Inc., Chicago, IL, United States). The mean ± standard deviation (SD), Kolmogorov‒Smirnov test to examine normality of distribution, and Levene’s test to examine homogeneity of variance were determined for all outcome measures. Changes in biochemical parameters and physical fitness parameters were compared between subjects over four time points for the HBOT and CON group using a repeated measures analysis of variance (RM-ANOVA). Finally, to evaluate the magnitude of the observed differences, the effect size was calculated (*eta squared*, η^2^) and interpreted as follows: <0.2 (small), >0.2 and <0.8 (moderate) and >0.8 (large) (Cohen, 1988). A power analysis was conducted using G^*^Power based on an effect size of 0.63 for CK levels from a similar study (Qu et al., 2024). With an α level of 0.05 and a desired power of 0.80, the analysis indicated that a total sample size of 22 participants would be required to detect statistically significant differences between the HBOT and CON group.

## 3 Results

**FIGURE 2:**
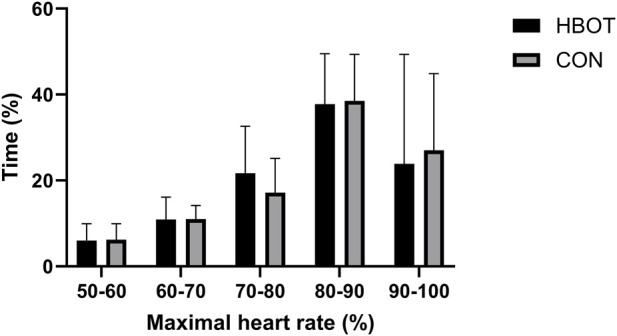
Time spent (%) in various heart rate zones as a percentage of maximum heart rate (HRmax) during the match. Note: comparisons are presented as HBOT vs. CON.

The main characteristics of the participants (height, weight, fat mass, muscle mass and body mass index (BMI)) as well as time spent (%) in various heart zones during the match are summarized in Table 1 and Figure 2.

**TABLE 1:** Descriptive statistics of the main characteristics of the participants.

Variables	HBOT	CON
Height (cm)	181.4 ± 5.1	178.2 ± 6.6
Weight (kg)	71.0 ± 9.5	66.9 ± 6.5
Fat mass (%)	12.4 ± 3.1	13.2 ± 3.9
Muscle mass (%)	43.6 ± 1.7	43.7 ± 2.2
BMI (kg/m^2^)	21.6 ± 2.6	21.1 ± 1.7

Legend: BMI, body mass index; HBOT, hyperbaric oxygen therapy group; CON, control group.

### 3.1 Biochemical parameters

**FIGURE 3:**
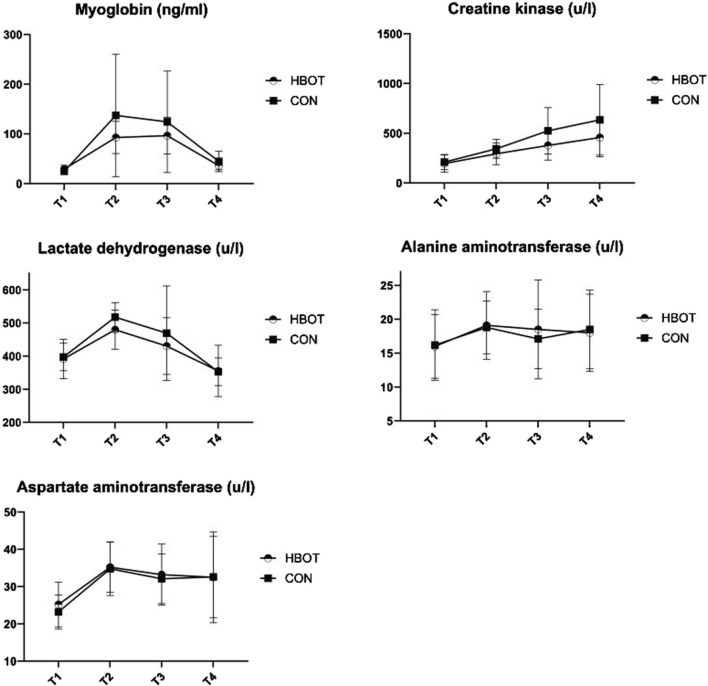
Biochemical parameters measured throughout four time points: pre-match (T1), post-match (T2), 1 hour after HBOT (T3) and 12 h after HBOT (T4).

Table 2 and Figure 3 shows descriptive data for both groups through four time points. The results indicated no significant differences between the HBOT and CON group in biochemical parameters at T1. Furthermore, there was no interaction effect between time and group for any of the measured variables (*p* > 0.05).

**TABLE 2:** Results for biochemical parameters.

Serum samples	T1	T2	Δ T2 vs. T1	T3	Δ T3 vs. T2	T4	Δ T4 vs. T2	*p*-value, η^2^
MB (ng/mL)								
HBOT	29.3 ± 7.9	92.9 ± 32.4	+218%	96.7 ± 36.9	+4%	36.3 ± 8.5	−61%	Group: *p* = 0.783, η^2^: 0.004 Time: *p* < 0.001, η^2^: 0.459 Interaction: *p* = 0.591, η^2^: 0.020
CON	24.9 ± 5.5	137.4 ± 123.2	+451%	124.4 ± 102.1	−9%	44.9 ± 20.8	−67%	
CK (u/l)								
HBOT	195.5 ± 86	294.2 ± 110.2	+51%	378.0 ± 147.0	+28%	457.6 ± 194.8	+55%	Group: *p* = 0.881, η^2^: 0.001 Time: *p* < 0.001, η^2^: 0.504 Interaction: *p* = 0.660, η^2^: 0.013
CON	210.3 ± 75.9	344.8 ± 95.9	+63%	526 ± 232.6	+53%	635.5 ± 354.5	+84%	
LDH (u/l)								
HBOT	391.5 ± 59.5	480.1 ± 59.2	+23%	430.6 ± 85.5	−12%	355.5 ± 77.5	−35%	Group: *p* = 0.439, η^2^: 0.034 Time: *p* < 0.001, η^2^: 0.568 Interaction: *p* = 0.484, η^2^: 0.036
CON	397.6 ± 41.4	517.9 ± 43.8	+30%	469.4 ± 142.4	−9%	352.9 ± 41.6	−31%	
ALT (u/l)								
HBOT	16.0 ± 4.7	19.1 ± 4.9	+19%	18.5 ± 7.3	−3%	18.0 ± 5.7	−6%	Group: *p* = 0.913, η^2^: 0.001 Time: *p* = 0.001, η^2^: 0.270 Interaction: *p* = 0.489, η^2^: 0.042
CON	16.2 ± 5.2	18.8 ± 3.9	+16%	17.1 ± 4.4	−9%	18.5 ± 5.8	−2%	
AST (u/l)								
HBOT	25.2 ± 5.9	35.2 ± 6.6	+40%	33.2 ± 8.2	−6%	32.5 ± 12.2	−8%	Group: *p* = 0.793, η^2^: 0.004 Time: *p* < 0.001, η^2^: 0.542 Interaction: *p* = 0.754, η^2^: 0.011
CON	23.2 ± 4.6	34.8 ± 7.2	+50%	32.1 ± 6.6	−8%	32.6 ± 10.9	−6%	

MB, myoglobin; CK, creatine kinase; LDH, lactate dehydrogenase; ALT, alanine aminotransferase; AST, aspartate aminotransferase; HBOT, hyperbaric oxygen therapy group; CON, control group; T1 – pre-match; T2 – post-match; T3 – after HBOT; T4 – 12 h after η^2^ – partial eta, effect size.

The match induced a significant increase (T1 vs. T2) in MB (*p* = 0.003), CK (*p* < 0.001), LDH (*p* < 0.001), ALT (*p* < 0.001), and in AST (*p* < 0.001).

### 3.2 Performance parameters

The results indicated no significant differences between HBOT and CON group in physical fitness tests at T1. Furthermore, there was no interaction effect between time and group for any of the measured variables (*p* > 0.05) (Table 3).

**TABLE 3:** Results for physical fitness (performance) parameters.

Physical Fitness	T1	T2	T3	T4	*p*-value, η^2^
Linear speed at 5 m (s)					
HBOT	1.11 ± 0.07	1.1 ± 0.09	1.06 ± 0.08	1.05 ± 0.08	Group: *p* = 0.850, η^2^: 0.002 Time: *p* = 0.084, η^2^: 0.141 Interaction: *p* = 0.374, η^2^: 0.055
CON	1.11 ± 0.11	1.08 ± 0.05	1.06 ± 0.05	1.09 ± 0.05	
Linear speed at 10 m (s)					
HBOT	1.83 ± 0.09	1.83 ± 0.10	1.81 ± 0.09	1.80 ± 0.09	Group: *p* = 0.968, η^2^: 0.000 Time: *p* = 0.187, η^2^: 0.089 Interaction: *p* = 0.092, η^2^: 0.118
CON	1.81 ± 0.06	1.82 ± 0.06	1.79 ± 0.06	1.85 ± 0.07	
Linear speed at 20 m (s)					
HBOT	3.09 ± 0.10	3.12 ± 0.13	3.10 ± 0.10	3.13 ± 0.13	Group: *p* = 0.813, η^2^: 0.003 Time: *p* < 0.001, η^2^: 0.351 Interaction: *p* = 0.370, η^2^: 0.059
CON	3.09 ± 0.10	3.13 ± 0.10	3.10 ± 0.10	3.18 ± 0.12	
SJ (cm)					
HBOT	32.70 ± 5.04	34.70 ± 5.31	35.60 ± 4.78	29.10 ± 8.32	Group: *p* = 0.987, η^2^: 0.000 Time: *p* = 0.005, η^2^: 0.279 Interaction: *p* = 0.353, η^2^: 0.059
CON	31.30 ± 4.39	35.70 ± 3.10	34.30 ± 3.96	31.40 ± 3.20	
CMJ (cm)					
HBOT	34.40 ± 5.17	36.80 ± 4.99	36.80 ± 7.92	35.00 ± 7.24	Group: *p* = 0.432, η^2^: 0.037 Time: *p* = 0.002, η^2^: 0.258 Interaction: *p* = 0.304, η^2^: 0.068
CON	33.60 ± 3.81	37.50 ± 3.56	35.50 ± 3.55	31.20 ± 2.76	
CMJa (cm)					
HBOT	40.30 ± 4.66	41.50 ± 2.80	43.20 ± 7.55	41.50 ± 9.27	Group: *p* = 0.466, η^2^: 0.032 Time: *p* = 0.049, η^2^: 0.141 Interaction: *p* = 0.513, η^2^: 0.044
CON	38.90 ± 3.68	41.00 ± 5.23	41.50 ± 5.58	37.60 ± 6.52	

Legend: SJ, squat jump; CMJ, countermovement jump; CMJa, countermovement jump with arm swing; HBOT-hyperbaric oxygen therapy group; CON, control group; T1 – pre-match; T2 – post match; T3 – after HBOT; T4 – 12 h after HBOT; η^2^ – partial eta, effect size.

The match had no effect on most of the performance parameters except for SJ (T1 vs. T2; *p* = 0.012).

### 3.3 Hooper index

**FIGURE 4:**
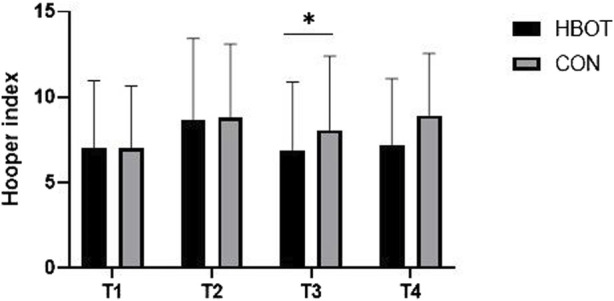
Hooper index (HI) values measured throughout four time points: pre-match (T1), post-match (T2), 1 hour after HBOT (T3) and 12 h after HBOT (T4). Note: comparisons are presented as HBOT vs. CON.

The results indicated no significant differences between HBOT and CON group in HI at T1. However, there was an interaction effect between time and group for HI (*p* = 0.012, η^2^ = 0.124). More precisely, the HBOT group had significantly lower HI values (8.6 ± 2.41) than the CON group (11.0 ± 3.23) at T3 (Figure 4). The match also presented a significant increase (T1 vs. T2) in HI (*p* < 0.001).

## 4 Discussion

The aim of this study was to determine the effect of a single session of HBOT on recovery parameters in elite youth football players. Briefly, the match clearly induced the anticipated biochemical changes indicating fatigue. However, the results indicated that there were no significant differences between the HBOT and CON group at T3 (after the intervention) for markers of muscle damage (MB, CK, LDH, ALT, and AST) and performance parameters (linear speed at 5 m, 10 m and 20 m, CMJ, CMJa, and SJ). Similarly, at T4, there were no significant differences between the groups for the same variables. Interestingly, the results revealed a significant difference between groups at T3 for HI, with a moderate positive effect of HBOT on this parameter.

Serum markers (MB, CK, LDH, ALT, and AST) have been extensively researched as parameters of muscle cell damage in elite football players (Ascensão et al., 2008; Ispirlidis et al., 2008; Meyer and Meister, 2011). The study’s findings revealed that MB, CK, LDH, AST, and ALT concentrations increased in both groups immediately following the football match. Although the concentrations of LDH, AST and ALT decreased after HBOT and continued in the same manner 12 h after HBOT, the results demonstrated no statistically significant effect of a single session of HBOT. Conversely, the most relevant marker of muscle damage, CK, had a drastic increase at all time points compared to baseline without a significant difference between groups. Existing studies (Branco et al., 2016; Harrison et al., 2001; Huang et al., 2021; Woo et al., 2020) examining post-exercise interventions have provided contradictory findings. While our findings are consistent with previous results showing no positive effect of HBOT on biochemical recovery parameters (Branco et al., 2016; Harrison et al., 2001; Huang et al., 2021), Woo et al. (2020) found that HBOT applied during the post-exercise recovery period is effective for treating exercise-induced muscle damage. It is noteworthy that none of these studies have assessed acute recovery using HBOT in elite youth football players immediately after a competitive match. Football is a very strenuous sport with high physiological and metabolic demands, where some markers of cell damage increase up to 72 h after the match and then return to their baseline values (Fatouros et al., 2010; Silva et al., 2013). More precisely, changes in direction and rapid accelerations and decelerations impose a significant eccentric load on the muscles, resulting in microinjuries and the release of CK and MB up to 72 h after a football match. In addition, their concentration depends on many factors, such as level of playing, period of season, individual responses, and previous activities (Brancaccio et al., 2007; Silva et al., 2013). In the present study, the participants were advised to refrain from stressful activities for 24h, which means that a certain concentration of CK remained in the blood. Furthermore, the participants evaluated in this study were in the off-season period, which probably resulted in higher concentrations of CK in the blood after the match. Therefore, our study indicates that a single 60 min session of HBOT may not be sufficient to improve biochemical recovery parameters in elite youth football players. The similarity in blood parameters between the HBOT and CON group could be due to the short duration and single session of HBOT, the natural and effective recovery processes in elite youth athletes, the potential insensitivity of the measured biochemical markers, or the timing of the measurements. To better understand the effects of HBOT, future studies should consider multiple sessions, a larger sample size, and possibly more sensitive or additional markers of recovery.

HBOT increases dissolved oxygen levels in the blood and results in a high partial pressure of oxygen in peripheral tissues, it could potentially stimulate the recovery process (Hodges et al., 2003). Since HBOT improves the oxygenation of skeletal muscles, it may accelerate the production of ATP in addition to the metabolic purification of metabolites that cause fatigue (Sperlich et al., 2017). Therefore, we hypothesized that HBOT treatment would positively affect the main physical fitness indicators of performance such as linear speed and vertical jump height. However, the findings of our study indicate no effect of a single session on these parameters when compared to post-match measurements in youth elite football players. One reason for this finding could be that our simulated game did not affect these parameters in the first place. In general literature describes a modest deterioration of jump and speed performance (Abaïdia and Dupont, 2018), although this is not always the case (Krustrup et al., 2010). Several factors, such as fitness level, game status (winning vs. losing), potential rewards and environmental conditions, indeed make these parameters variable (Abaïdia and Dupont, 2018). One could also argue that the lack of impact is caused by a too low intensity during the game, although this is not substantiated through our heart rate data and the biochemical changes that occurred. The lack of effect after HBOT in the current study is congruent with the work of (McGavock et al., 1999; Hodges et al., 2003), who found no significant effect of acute HBOT treatment on performance. More precisely, previous studies (Hodges et al., 2003; McGavock et al., 1999) tried to determine the acute effects of HBOT at 2.5 ATA for 90 min on cardiorespiratory fitness and concluded that the experimental groups did not improve or deteriorate performance on the maximal and submaximal running tests. These findings contrast with a previous study (Mihailovic et al., 2023) reporting post-HBOT treatment as an efficient way to improve power output during cycling. However, the different parameters and populations assessed within studies investigating the single-session effect of HBOT on post-exercise performance make it difficult to compare the results. Although several studies have examined the acute effects of HBOT on performance, to date, no study has specifically examined the acute effects of HBOT on several parameters of physical performance in elite youth football players, who exhibit fewer performance impairments and metabolic disturbances than lower-level players. More precisely, the large muscle groups involved in running or jumping become more resistant in this population due to intense training (Reilly et al., 2008). Also, young elite players (U20) have a shorter and more efficient recovery. Nevertheless, it is crucial to draw attention to the beneficial findings of a recent study (Burgos et al., 2016) investigating the effects of 3 weeks of HBOT training on oxidative stress markers and endurance in young football players. Specifically, the authors (Burgos et al., 2016) found that 3 weeks of HBOT did not cause an increase in oxidative stress but improved endurance capacity. Therefore, if utilized for a long period of time, HBOT may have significant ergogenic effects in professional football players.

Our results showed a significant positive effect in HI after the HBOT compared to CON 1 hour after the treatment. Since the HI parameter is the most widely used parameter in football for subjective evaluation of match-induced fatigue, this indicates that although we were unable to induce significant differences in biochemical or performance parameters, we were successful in reducing the perception of fatigue after the match. The HI has been employed as a tool to detect changes in wellbeing in football players, with specific focus on fatigue, stress, sleep quality and DOMS, over the course of a season (Silva R. M. et al., 2022) or in the early-season *versus* in-season (Silva A. F. et al., 2022). Furthermore, this confirms previous findings that the HI index is more sensitive than physiological parameters, such as heart rate variability (Rabbani et al., 2019) or blood parameters as measured in our study, to track fatigue in a specific football population.

The major strength of this study is that it was the first double-blind randomized controlled study investigating the effects of a single session of HBOT on biochemical recovery and performance parameters in elite youth football players. Furthermore, the evaluation of an extensive range of parameters and the protocol used are strengths to consider. However, some limitations of our study should be acknowledged when interpreting the findings. First, the findings should be interpreted with caution due to the specific pool of participants included, representing a small sample size because of the methodological constraint of employing one football game and the size of the Barox HBOT chamber. Given the actual sample size in this study (n = 20), the study may have been underpowered to detect medium effect sizes for other outcomes. Second, the studies’ comparison with current literature is difficult because of the limited number of studies investigating the acute effects of a single session of HBOT. Third, we advised the participants to refrain from any form of physical activity for a period of 24h, but we did not monitor or control their activities during the period of 48 h. Finally, although elite football players were recruited in our study, our findings are not generalizable to senior male professional football players, female football players, or athletes competing in other sports. Our research does not support the effectiveness of a single session of HBOT as a recovery model due to the absence of significant effects and the cost of treatment and equipment. However, large cohort studies examining acute effects on male and female football players and athletes competing across a wider range of sports should be conducted in the future. In addition, future research will have to further investigate acute HBOT protocols of different durations. Finally, future research will have to concentrate on the effects of sequential HBOT sessions on recovery and performance parameters.

## 5 Conclusion

In summary, our findings showed that a single session of HBOT did not have significant effects on selected biochemical recovery and performance parameters in elite youth football players. We did observe a lower HI score after the HBOT session compared to the placebo group, suggesting that HBOT may have a moderate positive effect on perceived recovery and wellbeing. Nevertheless, our findings may suggest a need for multiple HBOT sessions or a larger sample size to observe significant changes. Future studies are needed to confirm or refute our findings and to determine the optimal use of HBOT as a recovery intervention in football.

## Data Availability

The raw data supporting the conclusions of this article will be made available by the authors, without undue reservation.
